# Biosensors for Biogenic Amines: A Review

**DOI:** 10.3390/bios11030082

**Published:** 2021-03-13

**Authors:** Helena Vasconcelos, Luís C. C. Coelho, Ana Matias, Cristina Saraiva, Pedro A. S. Jorge, José M. M. M. de Almeida

**Affiliations:** 1 School of Agrarian and Veterinary Sciences, University of Trás-os-Montes and Alto Douro, 5001-801 Vila Real, Portugal; helena.s.vasconcelos@inesctec.pt (H.V.); crisarai@utad.pt (C.S.); 2 INESC TEC—Institute for Systems and Computer Engineering, Technology and Science and Faculty of Sciences, University of Porto, 4169-007 Porto, Portugal; lcoelho@inesctec.pt (L.C.C.C.); ana.b.teixeira@inesctec.pt (A.M.); pedro.jorge@inesctec.pt (P.A.S.J.); 3 Department. of Physics and Astronomy, Faculty of Sciences, University of Porto, 4169-007 Porto, Portugal; 4 Department of Physics, School of Science and Technology, University of Trás-os-Montes and Alto Douro, 5001-801 Vila Real, Portugal

**Keywords:** biogenic amines, food storage, biosensors, food spoilage, shelf-life

## Abstract

Biogenic amines (BAs) are well-known biomolecules, mostly for their toxic and carcinogenic effects. Commonly, they are used as an indicator of quality preservation in food and beverages since their presence in higher concentrations is associated with poor quality. With respect to BA’s metabolic pathways, time plays a crucial factor in their formation. They are mainly formed by microbial decarboxylation of amino acids, which is closely related to food deterioration, therefore, making them unfit for human consumption. Pathogenic microorganisms grow in food without any noticeable change in odor, appearance, or taste, thus, they can reach toxic concentrations. The present review provides an overview of the most recent literature on BAs with special emphasis on food matrixes, including a description of the typical BA assay formats, along with its general structure, according to the biorecognition elements used (enzymes, nucleic acids, whole cells, and antibodies). The extensive and significant amount of research that has been done to the investigation of biorecognition elements, transducers, and their integration in biosensors, over the years has been reviewed.

## 1. Introduction

With the expected increase in the human population, there is a need for new sustainable sources of raw materials, as well as new methods of food preservation, so food security appears as an extremely important requirement to ensure that along the food chain they are not contaminated by physical, chemical or microbial agents, in addition to viruses and parasitic prions that are capable of causing disease when ingested [1].

The world health organization estimates that more than 200 diseases are caused by eating food contaminated with bacteria, viruses, parasites, or chemicals [2]. This number may be higher because many diseases caused by food intake are not being reported, as well as the difficulty of establishing a cause-effect between eating contaminated food and illness or death. Therefore, it is very important to ensure that all the consumed food is not contaminated throughout the entire food chain with agents that can cause harm. Food can be contaminated in different stages of its processing, such as production, preparation, packaging, distribution, and consumption [3].

Food poisoning is generally caused by parasites, viruses, and bacteria, but poisoning can also be caused by chemical or natural toxins, such as aflatoxins, mycotoxins, and biogenic amines (BAs) [3].

Knowledge and detection of BAs in foods is important because these compounds at certain levels can cause neurotransmission disorders, due to their action as fake neurotransmitters, such as nausea, headaches, and palpitations, especially if some monoamine oxidase (MAO) inhibitors are also ingested, such as drugs or alcohols. The effects caused by the high ingestion of these compounds may be more adverse in sensitive consumers having a reduced mono and diamine oxidase activity, the enzyme responsible for its detoxification [4]. In addition, BAs are considered as precursors of carcinogens such as *N*-nitrosamine compounds [5].

The most used techniques for detecting BAs in foods are chromatographic techniques such as capillary electrophoresis (CE), gas chromatography (GC), thin-layer chromatography (TLC), and high-performance liquid chromatography (HPLC) [6]. Although the referred methods are widely used, they have some disadvantages such as the use of expensive equipment, specialized people are required to use the equipment, and pretreatment and derivatization of complex samples and time-consuming. In the last few years, many studies in which the authors resort to the use of biosensors (BSs) for the detection of BAs have been published. They are considered sensitive and robust devices that offer a simple, economical, and fast alternative in the detection of BAs [7].

## 2. Biogenic Amines

Biogenic amines are nitrogen compounds of low molecular weight with biological activities and can be found in several food products with variable concentrations, including fish and fish products, meat and meat products, dairy products, soy products, fermented vegetables, and alcoholic beverages, such as wine and beer [8,9].

Being used as indicators of food quality, they present high resistance to temperature treatments making them more difficult to eliminate [9].

The most common BAs found in food are putrescine (PUT), cadaverine (CAD), spermine (SPM), spermidine (SPD), β-phenylethylamine (PHE), histamine (HIS), tyramine (TYM), trimethylamine (TMA), tryptamine (TRY) and agmatine (AGM) [5,10]. Depending on the number of amine groups, biogenic amines are classified into monoamines (PHE and TYM), diamines (CAD, PUT, and HIS), or polyamines (SPM and SPD) [10].

They are formed and degraded through several pathways during the metabolic processes of plants, animals, and microorganisms [11], namely, by the microbial decarboxylation of amino acids which is dependent of the level of decarboxylase activity, specific bacterial strains present and the availability of the amino acid substrate [9]. Removal of the α-carboxyl group from an amino acid leads to the corresponding BAs [10]. Its formation can occur through a set of enzymatic reactions, as reductive amination, transamination, decarboxylation, and degradation of certain precursor amino compounds. However, the most common enzymatic reaction is the decarboxylation of amino acids and as these amines are formed by living microorganisms, are called biogenic [12]. Their formation by microorganisms requires some conditions, such as the presence of decarboxylase-positive microorganisms and conditions that allow bacterial growth, availability of free amino acids, decarboxylase activity, and decarboxylase synthesis [12].

**Figure 1:**
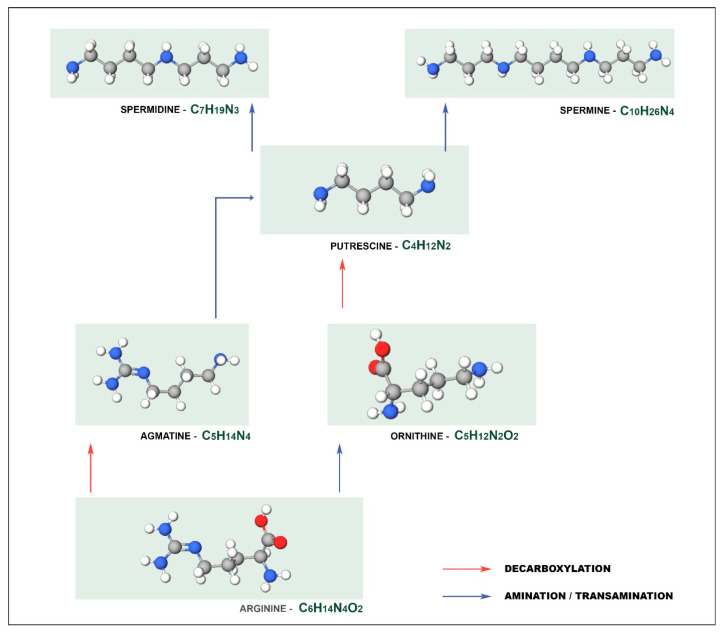
Different metabolic pathways for the synthesis of putrescine.

For example, PUT can be produced by different metabolic pathways, as illustrated in Figure 1, which can be synthesized either directly from ornithine by ornithine decarboxylase, or indirectly from arginine by arginine decarboxylase via [13] while SPD and SPM derive from PUT. From PUT, spermidine synthase catalyzes spermidine formation, which is a precursor of other polyamines such as spermine and structural isomer thermosperine [14].

Histamine is usually produced in species of the *Scombroidae* family such as mackerel and tuna, bluefish, mahimahi, or sardine and has been associated with scombroid poisoning. High HIS levels in this fish family have been explained by the naturally high free histidine, which is one of the amino acids that can be converted to intermediates of the tricarboxylic acid [15]. If there is only HIS, the metabolizing enzymes, such as diamine oxidase (DAO), the HIS metabolization process can occur, which can be inhibited in the presence of PUT and CAD. The amines PUT and CAD have been studied mainly as indicators of microbial deterioration and growth, while TMA has been found to be one of the principal volatile amines produced by spoilage bacteria [15]., reinforcing the importance of detecting BAs in food, assuring quality and freshness [16].

These compounds, when endogenous and naturally formed by animals, microorganisms and plants, are classified as polyamines and have important biological functions, for example, they play a significant role in the regulation of body temperature, in the cardiovascular system (blood pressure), in the nervous system (neurotransmitter), in the expression of regulatory genes, in the cell growth and differentiation, in the immune response, in gastric secretions and in inflammatory processes. In addition, they are nitrogen sources and precursors for the synthesis of alkaloids, hormones, proteins, and nucleic acids [12,17]. However, their presence in food is considered to be an indicator of food spoilage [18], because the occurrence of BAs in foodstuffs is caused by the metabolic production of contaminating microorganisms that induce undesirable organoleptic properties and adversely affect the taste and aroma of food [13]. The formation and concentration of BAs in food is influenced by several factors: raw materials (pH, composition, strength, ion), microorganisms (decarboxylase activity), and processing and storage conditions (cured, fresh, fermented, modified atmosphere, refrigerated) [17].

Histamine has been associated with scombroid poisoning in many studies; the most common symptoms of poisoning are vertigo, itching, faintness, the inability to swallow, and a burning sensation in the mouth [15]. Besides HIS, the BA with the most serious effects on human health is TYM, which has a vasoconstrictor effect and causes headache, nausea, increased cardiac, respiratory disorders, vomiting, and elevated blood glucose [19]. Its toxicity is familiar as “cheese reaction” because it was observed following the consumption of cheeses and fermented foods, where lactic acid bacteria (LAB) are mainly responsible for producing TYM, are present [19]. Regarding CAD and PUT alone, they have low toxicity but can increase the effects of HIS and TYM toxicity because they cause the inhibition of metabolizing enzymes [19].

## 3. Biosensors

Several analytical approaches have been elaborated for the quantitative analysis of BAs such as capillary electrophoresis (CE), gas chromatography (GC), ion-exchange chromatography (IEC), high-performance liquid chromatography (HPLC), and thin-layer chromatography (TLC) [7]. However, these techniques have some disadvantages, such as skilled personnel required to operate, long period analysis and sample pretreatment and derivatization. Contrary to all these methods, biosensors are robust, innovative, sensitive, and automated devices that offer rapid, simple and cost-effective solutions for the detection of BAs [7].

**Figure 2:**
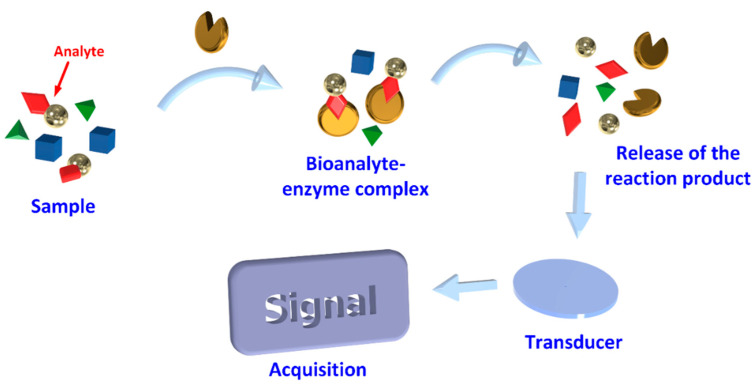
Schematic diagram of the basic principles of the biosensor.

Biosensors are devices that comprise biological components, which upon recognition of specific target analytes, a signal is produced that is then amplified, processed, and converted into a digital format [7] as shown in Figure 2. Such structures are accomplished by using specific biochemical reactions mediated by immobilized enzymes, immunosystems, tissues, organelles, or whole cells to detect chemical compounds. The supporting substrate may be silicon, polymers, glasses, polymethyl methacrylate, polydimethyl siloxane, among others, coated with a buffer layer, such as silicon nitrite, silicon dioxide, metals, and metal oxides [20]. These are then coated with biorecognition molecules that selectively capture target molecules such as enzymes, antibodies, and DNA or RNA molecules. The output of a Biosensor (BS) is a signal usually electrical, magnetic, mechanical, thermal or optical. A detection system compatible with the sensor platform must then be designed [20]. According to their interaction with the analyte, they can be divided into biocatalytic receptors, which are based on catalytic reactions, bio-affinity receptors based on substrate specificity, and hybrid receptors based on complementary sequences of DNA or RNA [21].

The transducer is an important component of a BS since it transforms the resulting biochemical signal into a quantifiable electronic signal [21], proportional to the analyte concentration [7]. The choice of transducers will be dependent on the type of signals emitted by the bioreceptor [21].

### 3.1. Biosensor Main Characteristics

Four main characteristics should be considered when a BS is being developed, such as selectivity, sensitivity, reproducibility, and reusability. In terms of selectivity, a BS should be very specific and be able to generate a positive result only in the presence of a certain target analyte, thus avoiding false-positive results [22]. In the case of BAs, a simpler approach consists of the decomposition of the matrix with a well-studied chemical compound. The specific reaction between an enzyme or an antibody with the appropriate target can be used to enhance BS selectivity [22]. Specifically, for BAs determination using biosensors, higher selectivity is required to overcome interferences. The complex matrix of foods is rich in compounds similar to BAs, structure-wise, that can give a similar response, thus increasing the signal obtained. One easy solution for this recurrent problem is to employ a conventional method like chromatography and seek validation of the biosensor [23]. Biosensors must be able to generate a response with small fluctuations in analyte concentrations. It is common to use the maximum (upper limit of detection) and minimum (lower limit of detection, LOD) analyte concentration that can be measured as figures of merit. It is important to consider the toxic limit associated with each Biogenic amine (BA) and use it as a reference to differentiate BS sensitivity from the one obtained by conventional analytical methods. Reproducibility is another main characteristic that defines a BS. The potential to give the same response to a target analyte when several nominally identical BSs are produced is of great importance [22]. Therefore, it should be reported reproducible variability among a set of BSs. Another important feature is whether the BS should be reused or not. Some authors say that disposable BSs are more popular due to the fact that deterioration of their elements in complex matrixes is avoided [22,23]. Moreover, the use of a BS several times will increase the overall analysis time, since the regeneration of the BS is required, i.e., the dissociation of the analyte from the binding site [22].

### 3.2. Biological Element

#### 3.2.1. Enzymes

When immobilized, they are more efficient in sensing than when in the free phase. In addition, enzyme stability over time increases after immobilization on the transducer surface [24]. One of the most important classes of enzymes used for BAs determination is amine oxidases. They catalyze the oxidation deamination of amines in aldehydes, in the presence of molecular oxygen (as an electron acceptor) with the production of ammonia and hydrogen peroxide.

Amine oxidase can be grouped in MAO, DAO, and polyamine oxidase (PAO). Their use in BAs quantification is dependent on substrate specificities. Despite their advantages, enzymes are difficult to extract, isolate, and purify, increasing their cost. They are either used in purified form or can be present in microorganisms or in slices of intact tissue [24].

Mediators like horseradish peroxidase (HRP) can be combined with enzymatic BS to overcome interferences and lack of sensitivity. This type of mediator is widely used as a dual system to determine BAs [25,26,27].The heme group from HRP endorses direct electron transfer from the redox center to the conduction sites available on transducers [7].

Using enzymes such as DAO and PAO along with several nanomaterials, such as carbon nanotubes, gold nanoparticles (AuNP), zinc oxide nanoparticles and platinum nanoparticles, in the fabrication of this type of sensors, has proven the enhancement of the electrochemical performance and catalytic efficiency, thus improving sensitivity to the target and preventing degradation [7,28].

A bienzymatic BS employing DAO and HRP for the detection of HIS in fish samples has been reported. These enzymes were co-immobilized into a polysulfone/carbon nanotubes/ferrocene membrane by means of a phase inversion technique onto screen-printed electrodes. The BS produced displays a sensitivity value as high as 1.9 × 10^7^ nA^−1^ and a LOD of 1.7 × 10^−7^ M [27].

The same enzyme was immobilized on a pre-activated immunodyne membrane using glutaraldehyde as a cross-linking agent. The sensor exhibited sensitivity to various levels of total BAs in the analyzed samples. However, the concentration must be in the range from 0.01 to 0.07 mg/mL; outside this interval, the sensor showed an anomalous behavior [29]. More recently, Omanovic-Miklicanin et al. developed an easy chemiluminescence one-shot BS for BA-determination in meat samples. Both DAO and PUO were employed and LOD obtained for those BS ranged from 0.8 to 1.3 mg/L [30]. It was possible to overcome monotonous preparation steps while adding a portable design to the BS with easy-to-use analytical equipment. In addition, disposable BS avoids the deterioration of their elements in complex matrixes. However, BS for both enzymes showed low stability when stored under low-temperature conditions, therefore, it remains a challenge for future work.

#### 3.2.2. Microorganisms

Microorganisms (e.g., bacteria and fungi) are incorporated in BS, since they can be used to detect molecules with higher specificity. It has been proven to be an interesting alternative to other biorecognition elements, and most importantly, applicable in environmental areas [31]. Because a cell is employed in this type of BS, the stability of the cell (i.e., optimization of factors such as sterilization, biocompatibility, among others) is its major limitation. Moreover, advantages of employing cells over enzymes in the sensor include overall better stability in terms of inhibitors, pH, and temperature conditions. Therefore, this cell-based BS is often preferred by the scientific community [32]. However, as far as we know, there are no published articles claiming the use of cells applied to the detection of BAs.

Recently, a BS for the detection of a bacteriophage using molecularly imprinted polymers (MIPs) to bind the target phage into the specific cavities on the electrode was described [33]. The method was also used for detection of E. coli in the concentration range 10^2^ to 10^7^ cfu/mL with a LOD value of 100 cfu/mL.

In another representative example, the bacteriophage C4-22 immobilized onto a rapid magnetoelastic substrate was used to detect Salmonella enterica serotypes in raw chicken samples. This sensor can perceive Salmonella concentration a low as 7.86 × 10^3^ cfu/mm^2^ [34].

It is a far less explored area when compared with other elements used in BS approaches. Nevertheless, it is possible to design and build a BS specific for BAs, with the help of genetic engineering simply by choosing cell type.

#### 3.2.3. Antibodies

Antibodies (Ab), often called immunoglobulin, are glycoproteins capable of identifying antigens (Ag) with high specificity, are commonly used as biorecognition elements. Immunosensors detect binding occurrences between Ab and Ag, with the formation of a stable complex substance. Either Ab or Ag can be immobilized on the surface of various types of transducers [35].

For the determination of HIS content in fermented vegetable juices, an immunosensor based on optical waveguide light mode spectroscopy (OWLS) was used [36]. The method consists of the conjugate of the Ag (histamine-bovine serum albumin) attached on the sensor surface with glutaraldehyde. The authors reported an LOD of 10^−2^ pg/mL. In this study, they also researched the relative specificity of the substrate of the antibody used, and for that purpose several BAs were applied. It was found that only PUT, CAD, and AGM responded to the sensor, and their responses remained moderate to mild, thus concluding that the competitive OWLS sensor is selective for histamine antigen.

Applying the principle of immunoreaction and total internal reflection of fluorescent it was presented recently an immunoassay produced by combining planar waveguide fluorescence for detection and quantification of aflatoxin M1 and melamine with a LOD of 0.045 and 13.37 ng/mL, respectively, which provided a new method for simultaneously determining different compounds such as aflatoxin M1 and melamine in milk products within 20 min [37].Competitive colorimetric immunosensor with detection limit 2 ng/mL was used to detect TYM in fish samples [38].TYM-bovine serum albumin was coated on a microplate and introduced as an analyze competitor. Inexpensive assays were achieved by incubating free TYM and horseradish peroxidase-labeled monoclonal anti-TYM Ab. The immunosensor gave significant analytical performance without the need for sample pretreatment [38]

Although the studies presented until now use different detection techniques, due to the low LOD reported, it can be concluded that the use of Ab as a biological recognition element presents high sensitivity.

#### 3.2.4. Nucleic Acids

Nucleic acids are biopolymers or small biomolecules, extensively used in the implementation of BSs, due to their inherent high specificity for recognition and ease of design [39]. Aptamers are short single-stranded DNA, RNA, or nucleic acid analogues, also called xeno nucleic acid molecules [31].

The following studies illustrate the use of small DNA structures for the functionalization of surfaces with totally different structural characteristics.

Queirós et al. developed a label-free DNA aptamer-based impedance BS for the detection of *E. coli* membrane proteins [40]. Two single-stranded DNA sequences were tested and these sequences were immobilized with and without the 6-mercapto-1-hexanol. Each step of the modification process was characterized by Faradaic impedance spectroscopy. The detection range ranged from 0.1 × 10^−6^ to 2 × 10^−6^ M.

Detection of thrombin based on aptamer binding using two different optical fiber-based configurations was shown by Coelho et al. [41]. Long-period gratings (LPFGs) coated with a thin layer of titanium dioxide (TiO_2_) as well as surface plasmon resonance (SPR) devices built on optical fibers coated with a multilayer of gold and TiO_2_. Both sensing systems, LPFG, and SPR-based were tested varying the concentration between 10 and 100 nm. The detection of 10 nm of thrombin was accomplished with a wavelength shift of 3.5 nm and a resolution of 0.54 nm.

The development of an aptamer-mediated colorimetric method for detection of chloramphenicol (CAP) residues in food was reported recently and the obtained LOD was 0.0031 ng/mL demonstrating a great recognition capacity of the aptamer to CAP. In addition, this aptamer-mediated CAP detection method was successfully applied to spiked food (fish and honey) samples [42].

A histamine-binding aptamer was selected by using SELEX (systematic evolution of ligands by exponential enrichment). The aptamer did not exhibit cross-reactivity with several other BAs studied. With this method, the authors managed to develop the detection of HIS in the urine based on a competition assay exploiting the H2 aptamer and magnetic beads reaching an LOD of 18 pm [43].

Another study was reported by the same authors, but in this case, the assay was based on the use of AuNP and the HIS aptamer H2 reported previously. This was applied to the analysis of samples of tuna and sardines in tomato sauce. The HIS assay for AuNP aptamer developed in this work is very sensitive, allowing the detection of 8 nm of histamine, being considered suitable for the analysis of food samples and clinical analysis [44].

Also, for detecting HIS, Dwidar et al. developed an aptamer of RNA that was converted into an aptamer of L-DNA for the detection of HIS in spoiled fish, reaching an LOD of 1 μm [45]. In addition, Valenzano et al. reported a study where they identify DNA aptamers that recognize TYM in the micromolar range [46]. Another biogenic amine detected by the same method was SPM, which is one of the indispensable components of biologically active cells and can regulate the physiological activities of the human body [47], however, its accumulation in the body can be toxic. Thus Tian et al. described the selection of sequences of aptamers for SPM by Capture-SELEX as well as the application of aptamers for the capture and detection of spermine in pork samples. This aptasensor exhibited a low detection limit of 0.052 nm [47].

From the studies presented, it was found that in addition to the low detection limits, the use of Aptamers also has other advantages, such as its ability to specifically bind and with high affinity to its target molecules, the ability to establish strong bonds and chemical stability.

The fundamental part of a biosensor is the choice of the biological element, since it is responsible for the recognition of the analyte of interest in the samples to be analyzed. Regarding the studies presented, the biological element still less explored in the detection of BAs is microorganisms. Despite its recognized advantages such as detecting a wide range of chemicals and a wide range of temperature and pH. On the other hand, enzymes have been one of the most used biological elements in biosensors for the detection of BAs, despite some disadvantages such as, difficult to extract, isolate, and purify, increasing their cost.

### 3.3. Immobilization of the Biological Element

**Figure 3:**
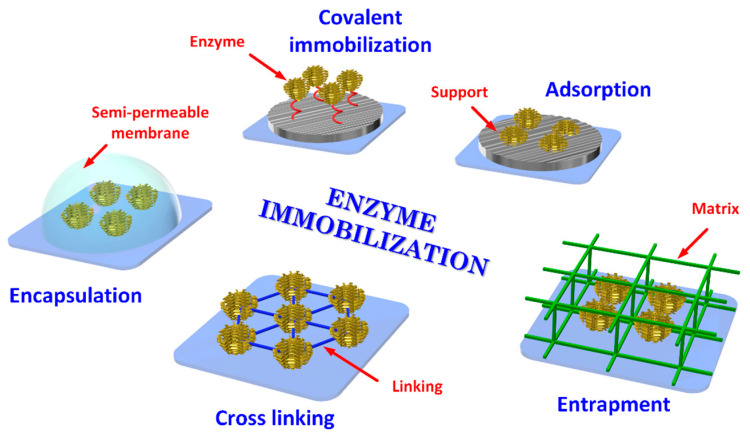
Typical enzyme immobilization processes.

Fabrication of a BS requires the immobilization of bioreceptors onto an appropriate substrate, which can be achieved by adsorption, entrapment (physical methods), covalence and cross-linking (chemical methods) [48,49] as illustrated in Figure 3. The immobilization structure may act as a support, or it may be a part of the signal transduction mechanism. The objective of the immobilization is to retain the activity of the bioreceptor’s elements on the surface of the transducer, therefore, the selection of an appropriate method depends on the nature of the bioreceptor, type of transducer used, physico-chemical properties of the analyte and operating conditions for the BS [50].

#### 3.3.1. Adsorption

Physical adsorption immobilization is due to van der Waals forces, hydrogen bonding, electrostatic or hydrophobic interactions [49] used, for example, for immobilization of microorganism cells [51].]. A suspension of microorganisms can be incubated with the electrode or with an immobilization matrix, followed by rinsing with a buffer solution to eliminate not immobilized cells. The method is also often used for the immobilization of DNA probes. For example, they can be immobilized on the surface of the working electrode via electrostatic adsorption between a DNA negatively charged phosphate group and the positive charged electrodes. Cationic polymeric films such as polypyrrole, poly-L-lysine, polyaniline, and polyethyleneimine have been reported for immobilization of DNA matrix through electrostatic adsorption [52].

#### 3.3.2. Self-Assembled Monolayer

Self-assembled monolayer (SAM) is a nanostructured thin film, 1 to 3 nm thick, that forms an aggregate due to chemisorption of organic molecules onto a certain substrate [7,53]. This aggregation is performed in order to achieve the ability of assembling individual molecules with different terminal groups, namely sulfides, thiols, amines, among others, into highly ordered structures for gaining a desired function [7,54].Such assembly can be achieved by controlling temperature and reaction with the substrate for a period of time [53].The substrate then is immersed in a dilute solution of the adsorbate [54], and the binding site of the molecule chemically reacts with the substrate and adsorbs to the substrate surface in the same direction, thus, forming intermolecular interactions (Van der Waals forces and hydrophobic interactions) between the adsorbed molecules where a monomolecular film with high density and high orientation is formed [53].Therefore, SAM formation offers flexibility to manipulate the monolayer by changing functional groups, according to the desired application. SAM is a robust technique of immobilizing certain biomolecules in the proximity of the electrode [7].

#### 3.3.3. Covalent Binding

The strongest method of enzyme immobilization is through chemical bonds called Covalent binding. Covalent bonds are in general formed between side-chain-exposed functional groups of modified supports, resulting in irreversible binding and producing a high surface coverage [55]. Functional groups on the enzyme surface, like glyoxyl, epoxy or amino groups, can be utilized in the multipoint type of covalent immobilization. For example, epoxy-groups can react with several groups on the surface of proteins like amino, thiol, phenolic, and imidazole. The amino groups of the enzyme can react with the carboxyl groups on the analyte [56].

In covalent binding, the synthesized DNA probe is in general linked with the group of thiols or amines at the end of 3′ or 5′ to bind covalently to the metal surface or to the specific functional group deposited on the surface of the electrode [52].

#### 3.3.4. Entrapment

The entrapment method consists of the physical confinement of an enzyme within a polymer without disturbing its activity [57]. Entrapment is also described as physical restriction of an enzyme within a confined network or space. This method can improve mechanical stability and reduce enzyme leaching and denaturation is generally avoided because the enzyme does not chemically interact with the polymer. The method permits create an optimal microenvironment for the enzyme that consists of matching the physico-chemical environment of the enzyme and immobilization material [58]. Entrapment by nanostructured supports like electrospun nanofibers and pristine materials was an important advance in immobilization with their wide-ranging applications in the field of fine chemistry, biofuels, and biomedicine BS [59].

#### 3.3.5. Cross-Linked

Enzymes are cross-linked to the support matrixes by intermolecular reactions using bifunctional reagents. They are immobilized firmly by covalent bonds, to improve stability and reusability. However, they can lose their catalytic properties during the cross-linking process. To immobilize enzymes, glutaraldehyde is the most commonly used substance [50].

Glutaraldehyde is a cheap and great cross–linker for use on commercial-scale operations. Dextran polyaldehyde has been successfully used in a few cases when glutaraldehyde presented bad performance. However, this method has been presenting poor reproducibility, low activity, and low mechanical stability. These issues are overcome by other protocols such as cross–linked enzyme aggregates (CLEAs), cross–linked enzyme crystals, and combi–CLEAs [60].

## 4. Transducing Methods

A large variety of transducing methods have been reported, such as electrochemical, optical and piezo-electric. The electrochemical method is usually further classified into three main categories based on the type of measurements, current (voltametric or amperometric), potential difference (potentiometry), and impedance (electrochemical impedance spectroscopy) [61].

### 4.1. Electrochemical Transducing

Electrochemical transducers are a wide class and are very promising in terms of autonomy, applicability, and output signal integration, either when used for screening of presence/default or for quantitative measurements. Signals are generated during biochemical reactions and are measured using suitable transducers. They are based on chemically modified electrodes where conducting or semiconducting materials are coated with a biochemical film [62,63]. Food matrices are known to have many electroactive interferents, with ascorbic acid being one of the most common interferents. Besides the presence of ascorbic acid in food matrices, uric acid is also reported to be an interfering species. However, the configuration and disposition of the biosensor can provide better selectivity, thus helping discard the possibility of interference [63]. Among the transducers that fall in this class, the amperometric, conductometric, impedimetric, and potentiometric types of sensors are further highlighted below.

#### 4.1.1. Amperometric Sensors

Amperometric transducers detect the electric current through an electrode when an electric potential is applied [64]. Their properties depend mainly on the physico-chemical characteristics of the materials employed in the transducer and of the enzyme immobilization methods [65]. Then some studies are presented in which the basis of BS is amperometry. These studies show the variety of applications of these devices ranging from the detection of BAs in blood samples from rats to food samples such as fish and pickled.

A BS for the determination of total BAs in rat blood samples has also been reported. Horseradish peroxidase immobilization on graphite was achieved by using bovine serum albumin, carbodiimide, and glutaraldehyde; the LOD was 17 ng/mL. This biosensor offers a good alternative to the existing methods, allowing rapid analysis and no pretreatment of the sample [25].

The determination of PUT and CAD using an amperometric arrangement has been carried out using a screen-printed carbon electrode with two working electrodes coated with MAO/tetrathiafulvalene and with MAO/gold nanoparticles. This BS showed a capability of detection of 9.9 and 19.9 ± 0.9 µM for PUT and CAD, respectively [66]. Another detection scheme using DAO, instead of MAO, entrapped by glutaraldehyde onto an electro-synthesized bilayer film was reported. For this sensor, the sensitivities obtained were 265.1 ± 2.2, 114.2 ± 3.0, and 57.5 ± 2.1 (nA/mM) for HIS, PUT, and CAD, respectively. The biosensor was successfully used in fish samples stored under correct and incorrect conditions [67]. HIS detection was recently reported where the authors developed an enzymatic sensor based on a screen-printed carbon electrode and DAO [68]. The enzyme was immobilized on the electrode surface through a cross-linking procedure with glutaraldehyde and bovine serum albumin. The LOD was 0.5 mg/L and showed that the sensor can detect HIS in real fish extracts. Also, to determine HIS in real fish samples, Pérez et al. proposed a bi-enzymatic structure using DAO and horseradish peroxidase [27]. The enzymes were co-immobilized into a polysulfone/carbon nanotubes/ferrocene membrane by means of phase inversion technique onto screen-printed electrodes, exhibiting a low LOD, 1.7 × 10^−7^ M. The functionalization with carbon nanotubes was also proposed for TYM detection [69]. The work was carried out using an amperometric-based BS on a modified screen-printed electrode carboxyl, functionalized with single-walled carbon nanotubes that was found to be a biocompatible matrix for immobilizing tyrosinase. The thick film electrodes of nanotubes were screen-printed by casting method followed by cross-linking with glutaraldehyde. The BS presented a LOD of 0.62 μm. This BS has been used successfully in determining TYM in pickled and smoked fish samples.

Biosensors should meet some requirements, among those being interference-free. Ascorbic acid interferences, as well as uric acid, were found in food products (e.g., kiwi, orange juice, and milk) [70]. In this study, a new electrochemical method for tryptamine determination using a paper-based microfluidic device and a thermoplastic electrode as an amperometric detector was developed. Interestingly, the authors have reported that the oxygen (compressed air) can oxidase ascorbic acid present at higher concentrations in an alkaline sample. This simple strategy seems to be effective when it comes to eliminating ascorbic acid peaks in the chronoamperogram of the amperometric sensors [70].

Interference characteristics of amino acids present in food matrices, in response to the amperometric biosensor, were measured. The selected amino acids of this study are naturally involved in BAs synthesis, and its possible interference on the biosensor response was evaluated by comparison of the signal obtained for 0.04 M BA standard solution with the signal obtained for solutions of the same concentration of those amino acids. It was conclusive that lysine and histidine amino acids present in the real sample changed the biosensor response for both DAO, and MAO [71]. Anti-interference properties should be further studied to evaluate the sensitivity of response of the biosensor developed.

For the immobilization of the enzymes referenced above (horseradish peroxidase, MAO, and DAO) used for the detection of PUT, HIS, CAD, and tyrosine, glutaraldehyde can be used, in combination with other agents, as an enzyme immobilizing agent on various types of electrodes. Particularly, for screen-printed electrodes, used as transducers, the right immobilization procedure must be adopted in order to improve the biosensor sensitivity and stability [72]. These studies show the variety of applications of these devices ranging from the detection of BAs in blood samples from rats to food samples such as cheese, raw, and pickled fish.

#### 4.1.2. Conductometric Sensors

Conductivity (reciprocal of resistivity) may change because of a specific biochemical reaction, leading to conductometric transducers which are based on the detection of conductivity. The method involves the conductivity determination of a sample solution between two parallel electrodes [73]. BS based on this phenomenon has important advantages: they do not need the use of a reference electrode; they operate at low-amplitude alternating voltage, thus preventing Faraday processes on electrodes; they are insensitive to light; they are suitable for miniaturization and large scale production using inexpensive technologies; they can be integrated using standard thin-film technology; the large spectrum of analytes of different nature can be determined on the basis of various reactions and mechanisms and the driving voltage can be sufficiently low to decrease the power consumption [74] significantly.

Biogenic amines, such as CAD, PUT, AGM, HIS, TRY, and TYM, were detected using a capillary zone electrophoresis method with conductometric detection. A clear separation of the six BAs with a detection limit (2–5 μm) was obtained [75]. Another approach for BAs detection was proposed by Sovovska et al., consisting of conductometric BS development based on calixarenes [76]. Thin-film interdigitated planar electrodes doped with a macrocycle as C-benzyl resorcinol calixarene, p-tert butylcalix [4] arene, or p-tert butylcalix [8] arene used for the development. The results showed an optimal concentration of calixarenes for the described BS membranes of 0.25 mg/mL.

Although the studies presented are not recent, from the studies presented, it was found that conductometric sensors have been used for the detection of BAs more associated with deterioration (PUT, CAD, and HIS).

#### 4.1.3. Impedimetric Sensors

Electrochemical impedance spectroscopy (EIS) combines the analysis of both the resistive and capacitive properties of materials based on the perturbation of a system at equilibrium by a sinusoidal excitation voltage signal. All substances within the electrochemical cell (such as resistors, capacitors, and inductors) present opposition to the movement of electrons and ions, resulting in a variation of the impedance (Z = V(t)/I(t)) [77]. A change in impedance of an electrochemical cell resulting from a redox biochemical reaction is measured as a function of frequency [78,79]. It is a measurement technique aiming to inspect electrode kinetics and electrode analyte binding characteristics. For EIS measurements, an alternate current, I(t), is applied and an electrical potential, V(t), is generated that has a phase difference in relation to the current [78].

The advantage of EIS is that the impedance of a biological reaction at the surface of electrodes can be inspected over a wide range of frequencies. A variety of biomolecules have been used as elementary detection elements of impedimetric BS with different degrees of success [79].

A sensor for TYM detection and quantification was developed by the immobilization of the enzyme tyrosinase in calcium phosphate materials followed by cross-linking with glutaraldehyde. Brushite cement-Polyphenol oxidase-glutaraldehyde-based BS led to a LOD of 4.85 × 10^−8^ M. The proposed biosensor was used to determine the TYM content in cheese samples [80].

A voltametric BS applied to the detection of HIS in-serum samples was studied and developed using MIPs incorporated into a carbon paste (CP) electrode as a MIP-CP electrode sensor platform. The LOD obtained with this sensor was 7.4 × 10^−11^ M [81].

To detect TYM a sensor based on a nanocomposite (polymer and gold NPs) film modified gold electrode was developed. It has been used in the detection of TYM in dairy products and fermented drinks. The LOD for this method was 0.04 µM [82]. In addition, for TYM detection, and using the same immobilization, but in this case for tyrosinase immobilization, the LOD was 0.71 µM higher than in the previous study [83]. Another study also showed a favorable perspective on the use of an impedimetric-based aptasensor for specific detection of HIS with an LOD of 7.80 mmol/L. The ability of these aptamers to bind histamine evidences the potential of these aptamers in applications such as histamine-specific biorecognition agents as well as to rapidly evaluate the histamine content in various food product samples [84].

From the examples presented above, the versatility of this type of BS is evident. Using the same detection principle, they can detect or quantify different BAs. In the studies summarized, different techniques were used; in one case calcium phosphate materials were used followed by glutaraldehyde cross-linking. In general, studies showed low LOD.

#### 4.1.4. Potentiometric Sensors

Potentiometry is based on the detection of the change in electric potential of an electrode when it comes in contact with a certain analyte [85,86]. These systems contain an indicator electrode and a reference electrode. Often potentiometric sensors are commercially available and they include glass coated and metal oxide electrodes, as well as ion-selective electrodes, which are a group of potentiometric sensors, the most widely used among them being a pH-sensitive glass electrode [87].

The signal is generated by charge separation at the interface between ion-selective membrane and the solution due to selective partitioning of ionic species between these two phases [86].

It is an appropriate technique for many applications because it allows the detection and quantification of various ions in wide ranges of concentrations and commercially available equipment can be used.

Potentiometric sensor for HIS detection in fish and wine samples was already reported, consisting of the incorporation of MIPs in poly(vinylchloride) membranes [88]. The LOD achieved was 1.12 × 10^−6^ mol/L. The authors concluded that the use of nanoparticles with high specificity and affinity allowed for label-free detection of histamine in real samples. To detect BAs in water electrodes functionalized with a SAM of 4-mercaptobenzoic acid as an artificial receptor for BAs was used. The carboxylate group can recognize the amine group through hydrogen-bonding or electrostatic interactions. The LOD obtained with this method was 25 mm [89].

A solid-state potentiometric sensor for the determination of TYR was optimized with the main purposed of using TYR alone as a food marker. Through this sensor optimization process, the effect of each factor was evaluated. Among those, interferences of several cationic species were evaluated by measuring the potentiometric selectivity coefficients with the separate solution method. The smaller the value, the better the selectivity to the target ion. These coefficients were found to be small enough, thus proving this potentiometric sensor selectivity towards TYR. Although all the examples of detection of BAs have as a principle the potentiometry, the studies showed different functionalization’s of their electrodes, conducting to different LODs.

### 4.2. Piezoelectric Sensors

Piezoelectricity-based sensors are an analytical technique able to record molecular interactions on an appropriate surface. They can be divided into two types, quartz crystal microbalance (QCM) and surface acoustic wave (SAW) devices. These sensors rely upon the measurement of changes in the frequency of resonance of piezoelectric crystals due to mass changes on the crystal surface [90,91]. In a QCM, an alternating voltage connected to the surface of the crystal by two electrodes causes mechanic oscillations of the crystal and its resonance frequency is then measured as the crystal is put into an oscillation circuit. The growth of a layer on the surface of the crystal results in a change of oscillation frequency, which is proportional to the mass deposited on the crystal [91].

In SAW devices, the higher modes of Rayleigh waves in the layered structures are generally called Sezawa modes, characterized by a guided wave, in which the acoustic velocity of the top piezoelectric layer is lower than that of the substrate or the layer below [92]. The application of an alternating electric field across the crystal substrate results in an alternating strain field. This causes a vibrational, or oscillatory, motion in the crystal, resulting in the generation of acoustic standing waves. The oscillator vibrates at a characteristic resonant frequency, depending on various parameters [93]. Crystals without a center of symmetry are typical materials with piezoelectricity properties [91].

As an example of using this technique, Mutlu et al. [94] describe the preparation of a quartz crystal sensor surface by plasma polymerization technique employing thiol and amine compounds, and characterization by contact angle of atomic force microscopy and modified surfaces by X-ray photoelectron spectroscopy, for each step. The authors concluded that the sensor can be used to detect HIS if there are no other interfering species. That is, if there are molecules with the same binding group, it can interfere with the detection of histamine, thus lowering the specificity of the sensor. The crystal surface was exposed to HIS solution and the frequency shift of the crystal was 575 ± 34 Hz. Detection of HIS was also accomplished by a SAW device together with MIPs. This sensor consists of an electrodeposited MIP film as the recognition element and a SAW crystal with Pt film electrodes as the signal transducer. The LOD was as low as 5 nm [95]. The combination of QCM and MIPs were was also reported for HIS detection and the authors synthesized a new MIP material for specific recognition by a sol-gel process and coated in a quartz crystal. The LOD was 7.49 × 10^−4^ mg/kg. The developed QCM sensor improved the efficiency of HIS detection when compared to the HPLC technique [96].

The studies presented refer to the two techniques used in piezoelectric sensors, such as QCM devices and SAW. The detection limits of these techniques were obtained in the order of mg/kg and nm, respectively, and despite different techniques, low LODs were obtained. Table 1 presents a summary of the studies previously described.

## 5. Optical Transducing

Optical-based BS are powerful detection and analysis tools. They can perform remote sensing and provide multiplexed detection within a single device. There are two main detection procedures that can be employed in optical biosensing: fluorescence-based detection and label-free detection [97].

Optical sensors have already been adopted in industry and in-field applications for the monitorization of various parameters. They have found application in several fields, such as food safety, environment, medicine, and biotechnology [98].

The biological sensing element is connected to an optical transducer system and the optical signal can be based on absorption, transmission, luminescence, reflectance, among others [99].

### 5.1. Surface Plasmon Resonance

Surface plasmons are coherent oscillations of free electrons that can exist at the interface between two media with opposite signs of dielectric permittivity, for instance, a metal and a dielectric medium. The electron motion creates electromagnetic fields outside, as well as inside the structure [100,101]. Surface plasmon resonance (SPR) based techniques have been used to study the interactions of biomolecules, such as enzymes, proteins, DNA, Ab, and Ag, due to its high sensitivity to the refractive index immediately adjacent to a thin metal film [100].

Localized surface plasmon resonance refers to an optical phenomenon generated by an electromagnetic wave trapped within conductive nanoparticles, typically, silver or gold. The phenomenon is the result of interaction between the incident electromagnetic wave and free electrons in the metal nanoparticles. This interaction produces coherent localized plasmon oscillations with a characteristic resonant frequency. The intensity and frequency of the plasmonic band are dependent on the shape, size, dielectric environment, composition, and distance of de nanoparticles [101].

Jiang et al. reported a detection scheme of HIS based on a SPR sensor and MIP films formed by spin-coating. This MIP-based SPR sensor shows a LOD of 25 μg/L [102]. However, in an older study, it was used as a SPR immunosensor for the analysis of HIS proposed by Y. Li et al. [103] where the LOD obtained was lower. The method is based on an indirect competitive reaction of an anti-HIS Ab in a sample solution where HIS is immobilized covalently on the sensor chip via alkanethiol; its LOD was 3 ppb.

For the detection of catecholamines, monoamines neurotransmitter, such as, dopamine, adrenaline, and L-Dopa a system consisting of an aqueous AgNO_3_ solution that includes polyvinylpyrrolidone (PVP), as stabilizer, in an alkaline medium was used. It was found that the reduction of Ag^+^ to silver nanoparticles (Ag-NPs) by the catecholamines in the presence of PVP produced SPR peak of Ag-NPs. The plasmon absorbance of the Ag-NPs allowed the quantitative spectrophotometric detection of the compounds with a LOD of 1.2 × 10^−6^, 9.7 × 10^−7^ and 8.6 × 10^−8^ M for the dopamine, adrenaline, and L-Dopa, respectively [104].

Contrary to the studies already presented that aim to detect BAs in the case of Y Li et al. [103], they present a study with the objective of testing the binding capacity of AGM and HIS. These compounds were immobilized on the surface of carboxymethylated dextran and their binding strength is assessed with oligonucleotides and plasmids isoforms. The authors concluded that the oligonucleotides and plasmid isoforms bind more strongly to HIS than to AGM.

From the examples presented, we can say that it is a technique with diversified applications since to detect the same compound you can use MIP, Ab, PVA, and Ag-NPs.

### 5.2. Surface-Enhanced Raman Spectroscopy

The surface-enhanced Raman spectroscopy (SERS) allows label-free, rapid, and non-destructive identification of different biological and chemical analytes [105]. It is essentially a molecular vibration spectroscopy technique, which can provide information on the structural characteristics of a molecule, resulting from an inelastic scattering process [106]. To improve its effectiveness, SERS can be combined with other techniques, such as chemical separation, colorimetry, biological capture, labeling techniques, and microfluidic devices. In addition, to obtain a more accurate characterization of samples, it can be combined with other chemical analytical techniques such as nuclear magnetic resonance and mass spectrometry, infrared spectroscopy, and X-ray photoelectron spectroscopy [106].

The detection of HIS in canned tuna was accomplished by combining MIP with SERS. This approach demonstrates to be a rapid and reliable technique to determine HIS at levels from 3 to 90 ppm [107]. Another alternative for detecting HIS was also measured in fish samples using SERS, but in this case, the sensitivity was improved by using widely available silver colloid SERS substrates. The method was tested in a concentration range of 0 to 200 mg/kg, according to the maximum limits established for this compound in fish [108].

Another example of the application of this technique for the detection of BAs was proposed by Chu et al. [109]. In this case, the authors used two techniques to detect HIS, contrary to what happened in the studies previously reported. The feasibility of determining HIS concentration in fish (*Miichthys miiuy*) by SERS) combined with density functional theory was presented. With this method, HIS at levels from 5 mg/kg to 400 mg/kg can be detected in fresh fish.

Another example of joining two techniques for HIS detection was proposed Xie et al. [110]. Here, in addition to the SERS technique, thin-layer chromatography (TLC) was used. The authors aimed at establishing a TLC-SERS method for the direct quantitation of HIS, visualized by fluram. Thus, by jointly using AgNPs and NaCl, an easy SERS method specific to in-situ derivatized HIS on TLC plates was established. With this method, it was possible to detect HIS concentrations of 54.3 ± 5.2 mg/kg, 69.5 ± 6.8 mg/kg for stored Ribbonfish and stored tuna, respectively.

For an improvement in the detection of BAs, this technique was combined with different techniques such as MIPs, density functional theory, and thin-layer chromatography.

### 5.3. Fiber Optic Biosensors

Fiber-optic biosensors (FOBS) use optical fibers as the transduction element and rely exclusively on optical transduction mechanisms for detecting target biomolecules [111]. Optical fibers can be categorized based on the structure, dispersion, refractive index profile, modes number, signal processing ability, and polarization [112]. They are made of a cylindrical core and a surrounding cladding. The core is usually doped with germanium to make its refractive index slightly higher than the cladding refractive index, which results in light propagation by total internal reflection. Light propagating through an optical fiber consists of two components: the guided field in the core and the evanescent [111].

These are used as transducers capable of detecting various analytes based on the variation of the refractive index caused by the concentration variation. The bare optical fiber is not sensitive or selective for chemical and biological sensing applications. Thus, it is necessary to apply functional materials integrated onto the optical fiber sensors to address this issue. The improved sensing and selectivity performance indicate the key role of the sensing materials in the development of FOBS [113].

Optical waveguides (or fibers) have been extensively studied to build optical BS, due to their small footprint, environmental ruggedness, high sensitivity, as well as remote sensing capabilities. Usually, such sensors are sensitive to the effective refractive index changes in the fiber surface [114]. To date, many fiber-optic refractive index sensors that are based on different detection strategies and configurations have been reported [114].

Regarding its application in the detection of BAs, it is still little explored. Below are examples of its application in the detection of BAs.

For the determination of HIS in fish and fish products, the authors used a continuous biconical tapered multi-mode optical fiber as a support to immobilize the enzyme DAO on chitosan and cross-linked with glutaraldehyde through enzyme multilayer assembly. The LOD of the FOBS was 15.8 ppm [115].

Alternatively, Pospiskova et al. [116] developed a FOBS with incorporated magnetic microparticles for DAO immobilization and can be efficiently used for BAs determination. The LOD for the BAs PUT and CAD are 25 and 30 μm, respectively.

Currently, there are still few studies on the application of optical fibers in the detection of BAs. However, due to the results obtained in the studies referenced with obtaining LOD in the order of μm, we can say that they can be a promising technique for the detection of these compounds.

## 6. Conclusions

Food security is a very important requirement throughout the food chain, so it is necessary to have a broad knowledge about the risks associated with food as well, as the processing and handling of them. Thus, ensuring that food is not a vehicle for disease transmission.

Considering the following subjects, namely food security and food safety, a more precise, sensitive, rapid, portable, and easy-to-use tool, which can detect BAs complex profile, is required. The techniques most used for the detection of BAs are chromatographic techniques such as, GC, CE, IEC, HPLC, and TLC. However, these techniques have some disadvantages, such as skilled personnel required to operate, and sample pretreatment and derivatization that require a large amount of reagents. Contrary to all these methods, biosensors are innovative, robust, and automated devices that offer rapid, simple and cost-effective solutions for the detection of BAs. This overview of BSs demonstrates that they are indeed the bioanalysis tool of the future, open the possibility to overcome existing problems such as detection limit, sensitivity, and specificity.

Regarding major accomplishments owing to BSs main characteristics, the lack of stability along with the high costs remains an unsolved challenge. For instance, enzymes used as biorecognition element seem to be an appropriate choice in a single shot BS due to their kinetics, since they are not consumed or altered during catalytic reactions and they can still be used multiple times, besides their cost is not very high since a few µL are required. To overcome low stability when stored, BS must be disposable and incorporated in a single-shot device. Optical biosensors emerge as a great candidate for BAs analysis, that is extensively dominated by the conventional chromatographic techniques. With such complex matrices in food chemistry, these are “immune” to electromagnetic interferences, therefore enhancing selectivity towards the analyte. Additionally, to make it possible for the BS to give an easy and sensitive answer in terms of analysis in the field, they should be able to have all the biosensing systems integrated on the same platform, where no pretreatment of the sample is required.

Biosensors are also very versatile because they allow the use of nanomaterials, functionalization with different compounds, and immobilization of enzymes, microorganisms, antibodies, and nucleic acids.

Currently, for the demanding food industry sector, BSs are intended to be devices that allow continuous monitoring with results in a few minutes without sample treatment.

Continuous focusing on obtaining reliable BSs can provide researchers as well as the public, in general, a better understanding of the impacts of BAs toxicity in food.

## Figures and Tables

**Table 1:** Electrochemical biosensors for biogenic amines determination.

Biosensor	Biogenic Amine	Recognition Element	Sample	LOD	Ref.
Amperometric	-	Enzyme	rat blood	17 ng/Ml	[25]
	PUT and CAD	Enzyme	octopus	9.9 ± 0.9 µm 19.9 ± 0.9 µm	[66]
	HIS, PUT e CAD	Enzyme	fish	265.1 ± 2.2; 114.2 ± 3.0; 57.5 ± 2.1 (nA/mm)	[67]
	HIS	Enzyme	fish extracts	0.5 mg/L	[68]
	HIS	Enzyme	fish	1.7 × 10^−7^ M	[27]
	TYM	Enzyme	pickled and smoked fish	0.62 μm	[69]
Impedimetric	TYM	Enzyme	cheese	4.85 × 10^−8^ M	[80]
	HIS	MIP	serum	7.4 × 10^−11^ M	[81]
	TYM	Gold NPs	dairy products and fermented drinks	0.04 µm	[82]
	TYM	Enzyme	dairy products and fermented drinks	0.71 µm	[83]
	HIS	Aptamer	solution at a physiological pH	7.80 mmol/L	[84]
Potentiometric	HIS	MIP	fish and wine	1.12 × 10^−6^ mol/L	[88]
Piezoelectric	HIS	Plasma polymerization	-	575 ± 34 Hz	[94]
	HIS	MIP	-	5 nm	[95]
	HIS	MIP	spiked fish products	7.49 × 10^−4^ mg/kg	[96]

## Data Availability

Not applicable.
